# An Unnamed Human Oral *Bergeyella* sp. as the Cause of an Unusual Bacterial Keratitis

**DOI:** 10.1155/2023/3288984

**Published:** 2023-05-25

**Authors:** Redion B. Petrela, Joshua A. Lieberman, Robert T. Swan

**Affiliations:** ^1^Norton College of Medicine, State University of New York Upstate Medical University, Syracuse, NY, USA; ^2^Department of Laboratory Medicine and Pathology, University of Washington, School of Medicine, Seattle, WA, USA; ^3^Department of Ophthalmology and Visual Sciences, SUNY Upstate Medical University, Syracuse, NY, USA

## Abstract

*Purpose.* We report a case of bacterial keratitis secondary to an undescribed *Bergeyella* sp. *Bergeyella* spp. are not easily cultured, and many reports have identified unculturable isolates through broad-range bacterial polymerase chain reaction (PCR). *Observations*. A healthy 29-year-old male was attempting to repair an acrylic cannabis water pipe when it shattered and a fragment hit him in the left eye. Two weeks later, he presented with foreign body sensation, scleral injection, and photophobia that were refractory to prolonged corticosteroid therapy. Following a subconjunctival triamcinolone injection, the patient developed a hypopyon and multifocal, midstromal, epithelized corneal infiltrates. Broad-range PCR of the aqueous fluid detected deoxyribonucleic acid closely matching the *Bergeyella* genus. Empiric treatment directed toward gram-negative bacteria led to the clinical resolution of the inflammation. *Conclusions and Importance.* This is the first reported case of ocular inflammation secondary to a *Bergeyella* spp.. As broad-range PCR testing becomes more accessible, we anticipate that additional PCR-positive and culture-negative scenarios will occur.

## 1. Introduction

*Bergeyella* spp. are aerobic, gram-negative rods that are uncommonly identified as a cause of human disease, most often presenting as cellulitis, bacteremia, and infective endocarditis [1]. Bergeyella spp. are not easily cultured [2], and many reports have identified unculturable isolates through broad-range polymerase chain reaction (PCR) [3–6].

We report a case of an unexpectedly indolent and refractory bacterial keratitis, initially believed to be noninfectious, that fully manifested after subconjunctival triamcinolone (TA) injection. Broad-range bacterial polymerase chain reaction (PCR) with sequencing of the partial 16S ribosomal ribonucleic acid (16S rRNA) gene performed on aqueous fluid detected deoxyribonucleic acid (DNA) from an undescribed member of the *Flavobacteriaceae* family. Subsequent molecular analysis indicated that the organism is an unnamed *Bergeyella* sp. found in the human oral cavity. Given the inability to culture the organism, empirical therapy was initiated and was successful. To the best of our knowledge, this is the first report of *Bergeyella* spp. causing inflammatory eye disease.

## 2. Case Presentation

A 29-year-old white male contact lens wearer was attempting to repair a cracked cannabis acrylic water pipe by applying tape in a tight, concentric fashion. This inadvertently resulted in it shattering with the impact to the left eye. The patient removed his contact lenses after the injury and did not reinsert them for the remainder of his care. Two weeks later he presented to an outside ophthalmologist with worsening foreign body sensation, blurred vision, photophobia, and injection of the left eye. Initial examination of the left eye revealed visual acuity of 20/150, 3+ scleral injection, and corneal stromal edema. The patient was placed on neomycin-polymyxin-dexamethasone drops and returned the next day for a TA injection (40 mg) into the sub-Tenon's space. Five days later, his symptoms and examination findings remained unchanged, and he was administered oral prednisone 100 mg daily. One month later, there was no improvement, and there was a concern for hypopyon at the limbus from 3-6 o'clock. The patient was referred to our institution for further evaluation.

**Figure 1:**
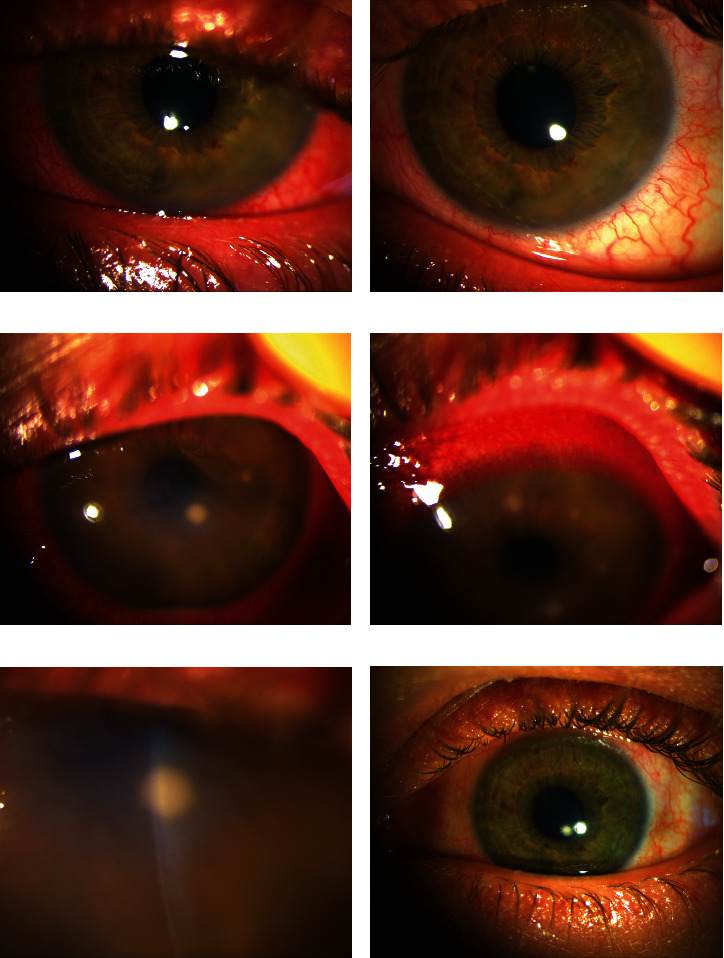
Keratitis caused by an unnamed human oral *Bergeyella* sp. (a) 2.5 months after onset with scleral injection and inferior nasal thickening of the cornea. Corneal epithelium was intact. (b) Incomplete blanching after phenylephrine 2.5% drops. (c, d) Six weeks following subconjunctival triamcinolone injection (2 mg in each quadrant). Corneal epithelium remained intact. (e) High magnification of the midstromal lesion. (f) Partial improvement 3 months after initiation of antibiotic therapy.

Two weeks later, 2.5 months after symptom onset, our initial examination revealed 3+ scleral injection with incomplete blanching after phenylephrine administration, uniform edema of the inferotemporal corneal stroma with several large inferior keratic precipitates, and 1+ cells in the anterior chamber with no visible hypopyon (Figures 1(a) and 1(b)). There was no corneal epithelial defect nor evidence of foreign body or penetrating injury identified on examination. Viral etiology was suspected, and the patient was started on a trial of acyclovir 800 mg five times daily in addition to his current regimen of topical prednisolone, topical cyclopentolate, and oral prednisone. Antibiotics were not restarted given a low suspicion for bacterial etiology in the absence of corneal epithelial defect or infiltrate and failure to improve previously on neomycin-polymyxin-dexamethasone drops.

A review of systems revealed poor dentition, no known history of herpes simplex infection, and no other systemic evidence of autoimmune disease. A serologic panel for autoantibodies revealed negative or normal results for antineutrophil cytoplasmic and antinuclear antibodies, antidouble-stranded DNA, rheumatoid factor, anticyclic citrullinated peptide, antiproteinase-3, and antimyeloperoxidase. Serologic studies were also negative or nonreactive for the following infections: *Treponema pallidum*, *Borrelia burgdorferi*, hepatitis B and C, human immunodeficiency virus, herpes simplex virus (HSV), cytomegalovirus, and *Toxoplasma gondii*. Urinalysis, C-reactive protein, sedimentation rate, and angiotensin-converting enzyme activity were within the reference range. The patient tested negative for human leukocyte antigen B27.

The patient failed to improve after two weeks of antiviral therapy. 2 mg of TA was injected into the subconjunctival space of each quadrant and oral corticosteroid was reduced. After receiving the injection, the patient reported rapid resolution of his discomfort and scleral injection, stopped all medications, and did not follow up as scheduled.

Six weeks later, the patient returned with left eye visual acuity of hand motion, 4+ scleral injection, 0.75-millimeter hypopyon (Figure 1(c)), and new circular stromal corneal opacities (Figures 1(c) and 1(d)). These opacities were midstromal with a flat appearance and mild adjacent corneal edema but otherwise clear overlying stroma and intact epithelium (Figure 1(e)). We presumed that discontinuation of therapy was responsible for recurrence, so the patient was restarted on topical and oral corticosteroids and oral antiviral therapy.

Two weeks later, the patient reported feeling more comfortable since starting the corticosteroid. On exam, the scleral injection and corneal stromal opacities were unchanged but the hypopyon was larger, measuring 1.25 millimeters. Given the mixed clinical picture, the possibility of an occult, indolent infection was considered. We decided to do additional testing but held on starting empiric antibiotic or antifungal therapy, instead favoring close follow up. Superficial culture and scrapings were felt to be low yield given the intact epithelium and clear stroma. While corneal biopsy could reach the midstromal location, this risked significant scarring and astigmatism. Instead, anterior chamber paracentesis was performed, and the sample was sent to the University of Washington for broad-range fungal and bacterial PCR analyses. While broad-range fungal PCR testing was negative, broad-range bacterial PCR targeting the V1-V2 region of the 16S rRNA gene [7] identified bacterial DNA in four of the four technical replicates. Sanger sequencing and BLAST analysis [8] identified the 315 bp product (after primer trimming; GenBank accession OP156817) as the *Flavobacteriaceae* family, following multiple layers of case review as previously described [9]. The sequence was unique in the clinical laboratory's experience and did not resemble common environmental or preanalytical contaminants, and controls were appropriately positive and negative. Given the genetic distance from firmly established species, the laboratory protocols precluded more specific identification. After receipt of these results, the patient was started on oral trimethoprim/sulfamethoxazole (TMP/SMX) twice daily, fortified amikacin drops every hour, polymyxin B/trimethoprim drops every hour, and atropine drops thrice daily. Given no view to the vitreous and the presence of scleritis and hypopyon, an intravitreal ceftazidime injection was administered. Corticosteroid and antiviral treatments were discontinued. A second anterior chamber paracentesis collected 0.2 milliliter of aqueous fluid for directed *Flavobacteriaceae* culture but no growth was observed after 5 days.

The patient noted increased comfort over the next few days and the hypopyon was absent on day seven. Given the improvement, therapy was continued with gradual resolution of the scleral injection and corneal opacities over the next three months. At his final in-person visit, his vision in the left eye was 20/30, the scleral injection was graded as trace, and the paracentral corneal stromal infiltrate was partially reabsorbed, with a moth-eaten appearance (Figure 1(f)). Antibiotic therapy was tapered slowly and discontinued. Due to the onset of the COVID-19 pandemic, his final visit was performed using telemedicine three months after the cessation of antibiotic therapy. The patient was comfortable, and the examination showed no scleral injection. The final visual acuity of the left eye was 20/40. Two years later, the patient has had no recurrence of inflammation.

**Figure 2:**
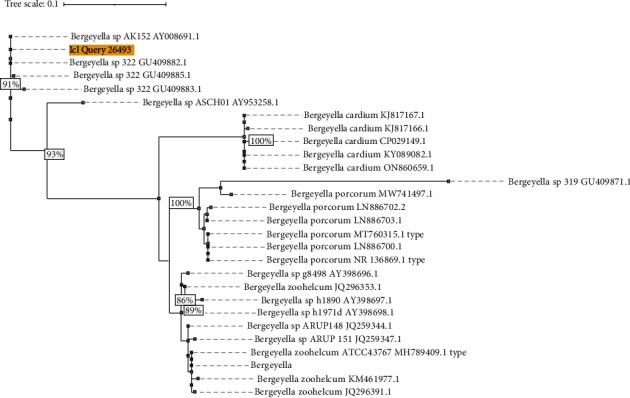
The phylogenetic tree includes 27 sequences representing the most closely related records in the Nucleotide database of the National Center for Bioinformatics, including all established *Bergeyella* spp. The patient sequence (highlighted, bold) forms a high-confidence clade (93% bootstrap) with unnamed human oral *Bergeyella* spp. and is quite distant from *B. zoohelcum*, *B. porcorum*, and *B. cardium*. Tree generated from the alignment of trimmed 16S ribosomal ribonucleic acid gene records and generated in IQ-Tree with generalized midpoint optimization and bootstrap branch support of 1000 ultrafast replicates; scale is substitutions per site.

In subsequent analysis, the sequenced product formed a high-confidence clade (Figure 2) with multiple *Bergeyella* spp. recovered from the human oral cavity, including 100% nucleotide identity to oral taxon 322 (GU409882.1) and AK152 (AY008691.1).

## 3. Discussion

The taxonomy of *Bergeyella* has evolved with the aid of 16S ribosomal gene sequencing. At the time of this patient's care, the genus *Bergeyella* was in the bacterial family *Flavobacteriaceae*, but it is now part of a related family, *Weeksellaceae* [10]. We report a unique case of sclerokeratitis caused by an unnamed *Bergeyella* sp., likely from the oral cavity, and describe the empirical treatment regimen that was curative for our patient. Our literature search revealed no case of ocular inflammation caused by *Bergeyella* spp.. Our patient was an otherwise healthy young male whose only risk factor was contact lens wear. However, it is unclear how the shattered cannabis water pipe precipitated these events. As its use involves water and oral contact, we speculate that it may have served as a reservoir for *Bergeyella* sp.. It is also possible that there was an undetected, clear, small, acrylic foreign body that breached the epithelium and served as the nidus for infection. Finally, the patient does have poor dentition, which could have been a source of hematogenous spread possibly seeding the site of impact injury. The role of the initial high-dose corticosteroids in creating an immunosuppressed state for *Bergeyella* sp. to propagate is unclear but presumed relevant, as the condition only fully manifested after a four-quadrant subconjunctival TA injection. Our empiric treatment regimen targeted toward gram-negative bacilli was aided by case reports showing improvement with TMP/SMX in two patients with *Flavobacterium* keratitis [11, 12]. We believe that the prolonged time to full resolution was due to scleral involvement.

Infections due to *Bergeyella* spp. are rare, often indolent, and frequently culture negative. Most reported cases involve bacteremia, endocarditis, or cellulitis [1, 3]. An intrauterine infection with an oral *Bergeyella* sp. closely related (99.37% sequence identity) to the pathogen in this patient has been reported with those authors hypothesizing that the source of infection was hematogenous spread from the oropharynx [4]. In that patient, blood and amniotic fluid cultures were negative, and *Bergeyella* was identified through amplification and sequencing of the entire 16S rRNA gene. Similarly, in another case report of *Bergeyella* cellulitis of the upper extremity, the organism was unable to be identified by culture, and the isolate was only able to be characterized by 16S rRNA gene sequencing [5]. Finally, in a case report of *Bergeyella* bacteremia and endocarditis, cultures from the surgical mitral valve specimen yielded no growth, and initial blood cultures misidentified the isolate as *Brevundimonas diminuta* [1]. 16S rRNA sequencing confirmed the identity of the organism, which revealed *Bergeyella zoohelcum* with 98.2% identity [1].

Given the fastidious nature of this species and these previous reports of failed cultures, it is not surprising that the same fluid that gave a positive PCR result was negative on subsequent culture. PCR has been shown to be effective in culture-negative cases for the identification of fastidious unculturable organisms and for the identification of organisms not previously regarded as responsible for infectious keratitis [13]. Even in the absence of culture growth, we do not believe that PCR was falsely positive because the sample was collected in a sterile manner from the anterior chamber, bacterial DNA was recovered in all technical replicates, the analysis passed multiple quality control processes in the laboratory, and antibiotic treatment appropriate for the identified organism led to clinical resolution. Additionally, the PCR product was a unique sequence in the 20+ year experience of the high-volume clinical laboratory. Therefore, we conclude that this organism is the etiologic agent and does not represent a preanalytical or environmental contaminant. The laboratory is not able to perform antibiotic sensitivity testing on direct samples (as opposed to isolates). At present, specific Clinical and Laboratory Standards Institute standards for antibiotic sensitivity for *Bergeyella* do not exist.

In addition to being the first reported case of *Bergeyella* keratitis, another unique aspect of our case was the use of anterior chamber fluid for diagnostic PCR. Typically, PCR of keratitis is performed via a direct swab of the exposed infiltrate [13–15]. We could not easily access the corneal infiltrates for direct culture, and so, diagnostic paracentesis was performed. Our literature search did not identify any publication primarily focusing on PCR of anterior chamber fluid for infectious keratitis, though success with this method has been reported in at least one patient [16]. It is possible that the PCR was positive because mild endophthalmitis was present. It is also positive that broad-range PCR is sensitive enough to detect pathogenic DNA in severe keratitis with a sterile hypopyon. As anterior chamber PCR may be diagnostically useful for atypical or refractory corneal infections, we endorse additional study on this subject.

Although *Bergeyella* belonged to the *Flavobacteriaceae* family at the time of this patient's care, it is currently assigned to the *Weeksellaceae* family [10]. There are several reports of keratitis caused by another member of the *Weeksellaceae* family, *Elizabethkingia meningosepticum* (*Flavobacterium meningosepticum* in earlier publications), as reviewed by Ang et al. [17]. Risk factors for reported cases include contact lens wear, trauma, and a persistent epithelial defect. *Elizabethkingia meningoseptica* is generally resistant to most antibiotics, although it is often susceptible to fluoroquinolones and TMP/SMX.

The treatment regimen directed toward gram-negative bacteria was effective in our patient, but it is unclear whether all components were necessary. Prior cases of invasive *Bergeyella* spp. infections were successfully treated with cephalosporins [1, 3]. We recommend treatment with a fortified cephalosporin and either topical gentamicin, fluoroquinolone, or fortified amikacin as an adjunct. We would recommend adding oral TMP/SMX, particularly if scleral involvement is observed.

In conclusion, our patient's bacterial keratitis was unique for having an inflammatory pattern that mimicked noninfectious, viral, and fungal etiologies. The presumptive etiological agent was identified as an unnamed human oral *Bergeyella* sp. through broad-range bacterial PCR testing of the aqueous fluid. As broad-range PCR testing becomes more accessible and common, we anticipate that additional PCR-positive, culture-negative *Bergeyella* spp. infections can be identified. Our experience provides guidance for an initial empirical treatment regimen in this situation.

## Data Availability

The sequence has been deposited in GenBank as record OP156817. The clinical data used to support the findings of this study are included within the article.

## Abbreviations

DNA: Deoxyribonucleic acid
HSV: Herpes simplex virus
PCR: Polymerase chain reaction
16S rRNA: 16S ribosomal ribonucleic acid
SUNY: State University of New York
TA: Triamcinolone acetonide injectable solution, 40 mg/mL
TMP/SMX: Trimethoprim/sulfamethoxazole.
